# Post-COVID-19 syndrome, who at risk and why: an insight from Qatar 2022

**DOI:** 10.3389/fpubh.2024.1368568

**Published:** 2024-04-12

**Authors:** Nada Adli, Mohamed Bala, Mohamed Iheb Bougmiza, Mohamed Ghaith Al-Kuwari, Abdul Hameed Al-Khenji, Nagah Selim

**Affiliations:** ^1^Specialist of Community and Preventive Medicine, Department of Community and Preventive Medicine, Hamad Medical Corporation (HMC), Doha, Qatar; ^2^Specialist of Community and Preventive Medicine, Department of Wellness Program, Primary Health Care Corporation (PHCC), Doha, Qatar; ^3^Community and Preventive Medicine Department, Primary Health Care Corporation (PHCC), Doha, Qatar; ^4^Community Medicine Department, Faculty of Medicine, Sousse University, Sousse, Tunisia; ^5^Senior Consultant of Community and Preventive Medicine, Primary Health Care Corporation (PHCC), Doha, Qatar; ^6^College of Medicine, Doha, Qatar; ^7^Public Health and Preventive Medicine, Cairo University, Cairo, Egypt

**Keywords:** post-COVID-19 syndrome, adults, Qatar, prevalence, persistent symptoms

## Abstract

**Background:**

Despite the recovery from the COVID-19 pandemic, many people experience post-COVID-19 syndrome, which negatively impacts their health and function. This condition has become a significant public health problem that requires immediate attention.

**Objective:**

To study the prevalence, clinical characteristics, and predictors of post-COVID-19 Syndrome in Qatar during 2022.

**Methods:**

An analytic cross-sectional study was conducted among COVID-19 confirmed cases from January 2022 to July 2022 in Qatar. A simple random sample was employed to include (*n* = 588) participants from the list of cases and invited to participate in a telephone survey. The World Health Organization’s standard case definition for PCS was adopted.

**Results:**

Out of 368, the prevalence of post-COVID-19 syndrome was 43.2% (*n* = 159). Most PCS cases were females (67.9%; *n* = 108), married (73.6%; *n* = 117), and university and higher educational level (83.6%; *n* = 133). However, 78.7% (*n* = 125) reported poor to moderate levels of social support. Only 30.2% (*n* = 48) of PCS patients had a history of chronic diseases, and 5.7% (*n* = 9) required hospital admission during acute illness. Among PCS cases, the most commonly reported symptoms were fatigue (75.5%), followed by anxiety (49.1%), forgetfulness (46.5%), mood alteration (45.3%), and general weakness (39.6%). The logistic regression revealed that female gender (AOR: 2.58 95%CI: 1.58–4.225, *p* < 0.0001), university and high educational level (AOR: 2.2, 95%CI: 1.256–3.98, *p* < 0.006), poor level of social support (AOR: 2.45; 95%CI: 1.55–4.13; *p* < 0.002), were significant predictors for PCS.

**Conclusion:**

Post-COVID-19 syndrome may go under-recognized. More efforts are needed to raise awareness and mobilize the resources to respond to this ongoing public health problem.

## 1 Introduction

Coronavirus Disease 2019 (COVID-19) is an infectious disease caused by the severe acute respiratory syndrome (SARS-cov-2) (1). People infected with COVID-19 may exhibit symptoms that vary in severity and take approximately 2 weeks to recover (2).

However, despite recovering from the COVID-19 infection, many patients experienced persistent symptoms that may last several months after the initial COVID-19 illness (3). These symptoms commonly include fatigue, shortness of breath, neuro-cognitive dysfunction, brain fog, anxiety and depression, and many other symptoms (4, 5), which may fluctuate or relapse over time. Patients with these symptoms still face difficulties in resuming their daily routines. This condition is now known as Post COVID Syndrome (PCS) (3).

Post COVID-19 Syndrome, commonly known as long COVID, is a complex multi-systematic disease that causes morbidities in people infected with COVID-19 (3). It can affect anyone exposed to SARS-CoV-2, regardless of age or severity of original symptoms (6). Initially, there was a lack of uniformity in Post COVID-19 Syndrome definitions within the scientific literature. However, the World Health Organization (WHO) defined the post- COVID-19 syndrome as a persistent physical, cognitive, and psychological set of symptoms that continue for 12 weeks and more following the acute illness and are not explained by any alternative diagnosis (3), and has been recognized as an international healthcare concern and an emergency-use ICD code has been issued (3).

It has been hypothesized that persistent symptoms after acute COVID-19 infection result from an immune-mediated disruption to the autonomic nervous system; it acts as an immune trigger like other post-viral autoimmune conditions (like Guillain Barré syndrome). This immune response, coupled with a lack of access to acute COVID-19 treatments offered only in a hospital setting, may explain why even those with less severe acute infections are still experiencing persistent symptoms (7–9).

Post-COVID-19 Syndrome (PCS) prevalence ranges from 5 to 80% globally, varying by region, socioeconomic factors, and follow-up duration based on a comprehensive systematic review conducted in 2021 (10). For example, PCS rates in Italy and Germany were 69 and 61.9%, respectively (11, 12), while in Spain and Belgium, they were 56.9 and 48% (13, 14). The incidence of PCS is estimated at 10 to 35%, with hospitalized patients experiencing rates as high as 85% (4, 15). In the United Kingdom, around 100,000 individuals have PCS, with 66% reporting impacts on daily activities (16). The Commonest symptoms reported after COVID-19 recovery included fatigue, dyspnea, sleep disorder, and difficulty concentrating, which persist at varying rates over time (17).

Furthermore, findings from systematic reviews and meta-analyses revealed several significant risk factors associated with post-COVID syndrome. These factors include Age, female gender, minor ethnicity, socio-economic deprivation, smoking history, obesity, and various comorbidities such as hypertension, diabetes, hypothyroidism, COPD, and asthma.

Additionally, admission to the intensive care units, invasive ventilation, and the duration of hospitalization were additional risk factors that may associated with post-COVID syndrome (18–20).

As the pandemic continues, many patients experience persistent symptoms and still face difficulties in resuming their daily routine despite recovering from the COVID-19 infection. Understanding the burden of this novel condition is imperative, with thousands of people at risk of developing PCS by the end of the pandemic.

To the best of our knowledge, there is a considerable research gap in the Middle Eastern and Gulf countries regarding PCS persistence symptoms and the long-term consequences on people’s health and productivity.

Given these considerations, our study endeavored to fill this knowledge gap by identifying the prevalence of this emerging disease in Qatar, delineating the persistent symptoms associated with PCS, evaluating the association between this syndrome and the socio-demographic and health-related characteristics, and contributing to the current knowledge regarding the magnitude and the potential predictors of prolonged COVID-19 infection.

## 2 Materials and methods

### 2.1 Study design and setting

An analytical cross-sectional study was conducted in Qatar in 2022. The setting was Qatar’s Business Intelligence Unit (BIU) at the Primary Health Care Corporation (PHCC), which is a record system that gathers, stores, manages, and transmits all patients’ electronic medical records across all primary health care centers (21).

The Primary Health Care Corporation (PHCC) is Qatar’s leading primary care provider, serving a population exceeding 1.6 million across 28 primary healthcare centers. During the pandemic, PHCC played a proactive role in offering COVID-19 testing at all primary health centers. Positive cases were promptly reported to the Business Health Intelligence (BIU) department within PHCC and subsequently communicated to the Ministry of Public Health (MOPH) (21).

This study met the PHCC’s guidelines for protecting human subjects concerning safety and privacy under protocol ID (PHCC/DCR/2022/06/31).

### 2.2 Participants sample size and sampling method

Since January 2022, the estimated number of people infected by COVID-19 was 15,000 based on CDC/PHCC-BIU statistics. Assuming a 43% prevalence of PCS (18, 19), a 95% level of confidence (CI) with an error rate of 5%, and a Design effect of 1, the required sample size was 368 participants based on OpenEpi® software version 3.01 using the following equation (22):


$$\begin{array}{l} \mathrm{Sample size}\;n=\\ \;\left[{\mathrm{DEFF}}^{*}\mathrm{Np}\left(1-p\right)\right]/\;\left[d^{2}/Z^{2}{{\;}_{1-\alpha /2}}^{*}\left(N-1\right)+p^{*}\left(1-p\right)\right] \end{array}$$

After adjustment for an anticipated 40% response rate to the telephonic interview, the total sample size was determined to be 588 participants.

All adults aged 18 years and above diagnosed with COVID-19 infection by either PCR or Repaid antigen test in Qatar from January 2022 to July 2022 and who could communicate in Arabic or English were eligible to participate in our study without any restriction on nationality or gender.

A computer-generated random numbers were used to select participants from the list (*N* = 588). The selected participants were contacted and recruited by telephone. Those who consented after understanding the study objectives were included and underwent telephone interviews, each lasting approximately 40–45 min.

### 2.3 Questionnaire development and validation process

This part was developed and constructed by the author after an extensive literature review. It contained 22 questions about Socio-demographic and health-related characteristics, which included information about age, gender, nationality, marital status, educational level, employment status, perceived monthly income, social support, and Household Crowding Index (HCI), in addition to the presence of co-morbidities, height, and weight, COVID-19-confirmation test, hospital and ICU admissions, the presence of persistent symptoms after recovery, and the duration of these persistent symptoms.

The face validity of the questionnaire was established and ensured by consultation with community medicine faculty and experts in the field. Translation validity was established by translating the English version into Arabic by two native Arabic speakers and then translating it back into English to ensure consistency. All authors then agreed upon the final version. Subsequently, the questionnaire underwent piloting on a convenient sample of 32 adults aged 18 years and above to assess its clarity, comprehensibility, and appropriateness.

### 2.4 Data collection tools and variables

Data was collected from the potential participants by trained interviewers through telephone-based-interview by used.

- Data extraction sheet: included the participant’s telephone number and the last confirmed COVID-19 infection date.
- And the socio-demographic characteristic and health-related Questionnaire.

### 2.5 Dependent variables

The dependent variable was the prevalence of post-COVID-19 syndrome, which was considered if the patient met the WHO standard case definition (had at least one persistent symptom 12 weeks or more after the initial infection with COVID-19 which cannot be related to any alternative diagnosis) (3).

Initially, the duration of post-COVID-19 symptoms was measured to the nearest (days/week/month), and later, it was categorized into two groups based on the case definition. Those who had persistent symptoms for 12 weeks or more were assigned to the PCS group and those who had persistent symptoms for less than 12 weeks after the initial infection were assigned to the non-PCS group (3).

### 2.6 Independent variables

The data collection tool was used to collect data about participants’ sociodemographic characteristics such as (age, gender, nationality, marital status, educational level, employment status, and perceived monthly income), which was assessed by a Likert scale and classified initially into four categories “More Than Enough, Enough, Just Barely Enough, Not Enough at All.”

In addition, the Household Crowding Index (HCI) was measured as the total number of Co-residents per household, excluding the newborn infant, divided by the total number of rooms, excluding the kitchen and bathroom. Then re-grouped into three categories: [<1, 1–2, and > 2]. The higher HCI indicated lower Socio-Economic Status (SES) (23).

Furthermore, we assess the social support level among participants using the Oslo Social Support Scale (OSSS-3), a validated and reliable tool for assessing social support. It consists of three items that evaluate the number of close confidants, the sense of concern from other people, and the relationship with neighbors, focusing on the availability of practical help. The total score of the OSSS-3 can range from 3 to 14, with lower scores indicating poor social support and higher scores indicating strong social support. The sum score on the OSSS-3 was categorized into three broad levels of social support: 3–8, indicating poor social support; 9–11, indicating moderate social support; and 12–14, indicating strong social support (21, 24, 25).

Regarding the health-related characteristics, we assessed the presence of co-morbidities by two categories (Yes vs. No), and the type of Co-morbidities such as (Hypertension, Diabetes mellitus, Asthma, etc.,) were listed for those who had history of co-morbidities. Additionally, information about height and weight were collected. The BMI was calculated and categorized into two groups (obese/overweight vs. Normal weight). Furthermore, hospital admission (Yes vs. No), and ICU admissions (Yes vs. No) were also assessed among all participants.

### 2.7 Ethical statement

This study was approved by the Primary Health Care Corporation Ethical Committee (PHCC-IRB); under protocol number (PHCC/DCR/2022/06/31). Verbal consent was obtained through telephone from each participant before the interview. The study was conducted in full conformance with the principles of the “Declaration of Helsinki” and Good Clinical Practice.

## 3 Data analysis

The database was constructed and analyzed using the Statistical Package for Social Sciences (SPSS)™ Software Version 25. Descriptive analysis was performed for the characteristics of the participants and data were presented as frequencies and percentages for categorical variables. Additionally, the normality distribution of the dataset was assessed using the Kolmogorov-Smirnov test. The utilization of either the mean ± standard deviation (S.D) or the median + Inter-Quartile Range (IQR) depended on the p-value obtained from the test.

A chi-squared test was used to identify significant associations between the Socio-demographic and health related characteristics and post-COVID-19 Syndrome. Logistic regression was also used to identify the independent factors of post-COVID-19 Syndrome and to calculate the adjusted odds ratios (ORs) with the 95% Confidence Interval (CI). *p*-values <0.05 (two tailed) were considered statistically significant.

## 4 Results

### 4.1 Sample characteristics

**Figure 1:**
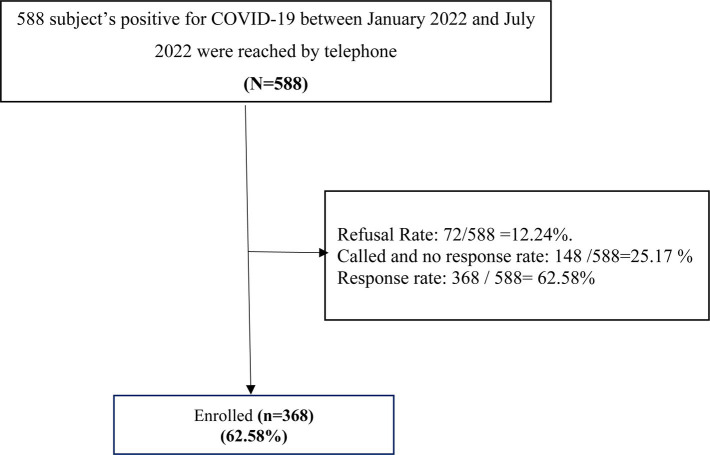
Flow chart of the study recruitment of the participants (*n* = 368).

During the data collection period, we approached 588 participants aged 18 years and above who had been diagnosed with COVID-19 infection between (January 2022 and July 2022) by telephone interview to participate in the study, giving a response rate of 62.5% as shown in Figure 1.

#### 4.1.1 Sociodemographic and health-related characteristics of the study participants

A total of 368 of the patients who were infected with COVID-19 in Qatar during 2022 are shown in Table 1. The Median age (IQR) of the COVID-19-infected adults was 36.0 [IQR: 30.0–43.7], and more than half of them (54.9% *n* = 202) were females. Most of the participants were non-Qatari (89.9%; *n* = 331), married (72%; *n* = 265), highly educated (76.6%; *n* = 282), and employed (79.6%; *n* = 293). In addition, more than half (58.4%; *n* = 215) of the adults infected with COVID-19 perceived enough monthly income, and 70.7% (*n* = 260) had a household crowding index between (1–2). Moreover, 73.1% (*n* = 269) of them had poor to moderate levels of social support.

**Table 1:** Frequency distribution of the sociodemographic and health-related characteristics of study participants infected with COVID-19 in Qatar during 2022 (*N* = 368).

Variable	Frequency (*n*)	Percentage (%)
**Age**
18–24	28	7.6
25–39	210	57.1
40 and above	130	35.3
**Median age (IQR)**	36.0 [IQR: 30.0–43.7]	
**Gender**
Male	166	45.1
Female	202	54.9
**Nationality**
Qatari	37	10.1
Non-Qatari	331	89.9
**Marital status**
Not Married*	103	28
Married	265	72
**Education status**
No formal education	3	0.8
Primary school	15	4.1
secondary education	68	18.5
University and Higher education	282	76.6
**Employment status**
Not-employed.	75	20.4
Employed	293	79.6
**Perceived Sufficiency of monthly income**
Not enough at all	74	20.1
Just barely enough	64	17.4
Enough	215	58.4
More than enough	15	4.1
**Household Crowding Index**
Less than 1	75	20.4
1−2	260	70.7
More than 2	33	9
**Level of social support**
Poor social support	116	31.5
Moderate social support	153	41.6
Strong social support	99	26.9
**History of chronic disease**
No	268	72.8
Yes	100	27.2
**BMI**		
Normal weight	96	26.1
Obese/overweight	272	73.9

*Not married = single, divorced, and widowed.

Regarding health-related characteristics, 27.2% (*n* = 100) of the study participants had a previous history of chronic diseases, and a significant proportion (73.9%; *n* = 272) were obese or overweight as shown in Table 1.

#### 4.1.2 Prevalence of post-COVID-19 syndrome and its related characteristics among the study population

**Figure 2:**
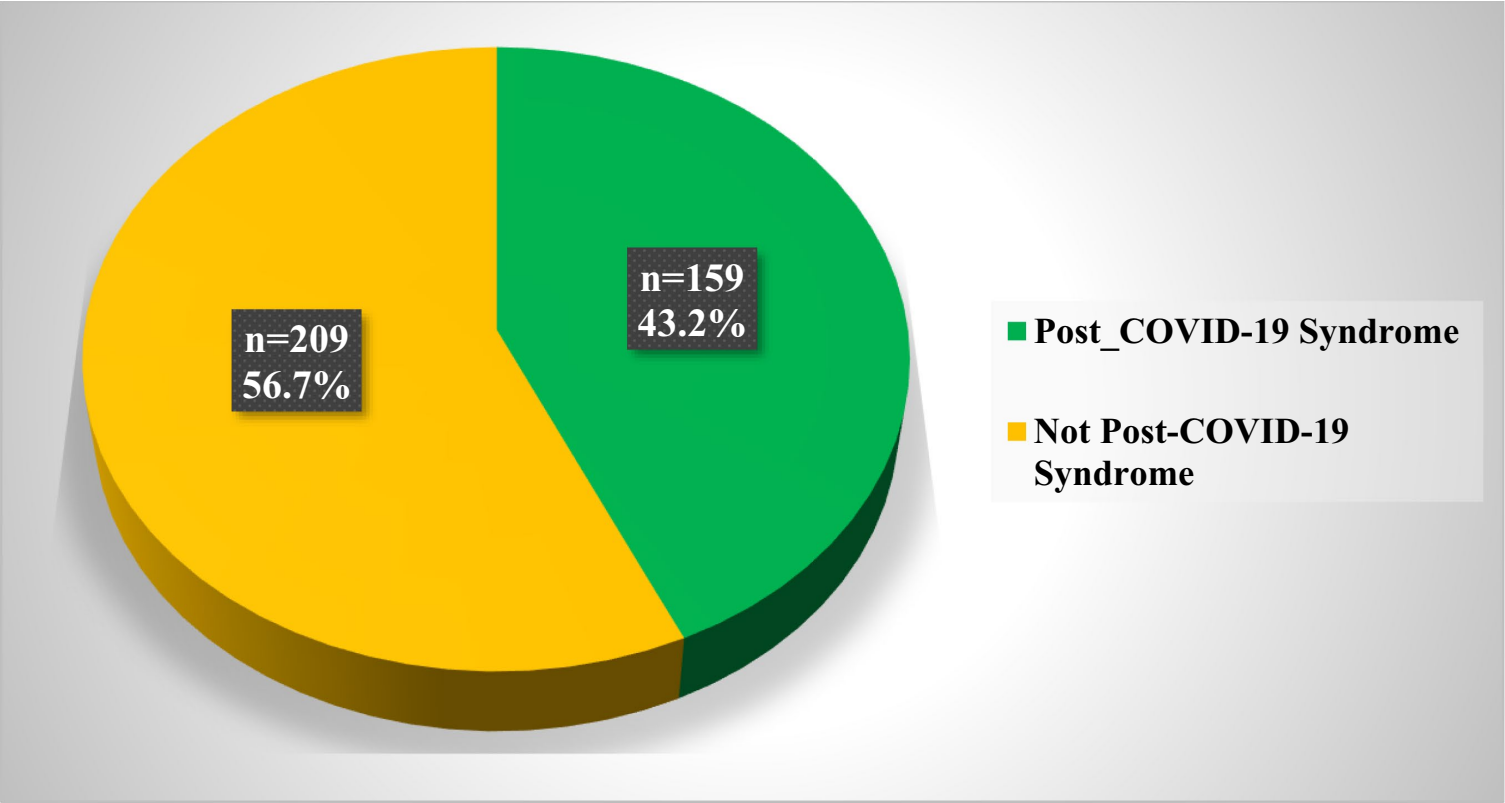
Prevalence of post-COVID-19 Syndrome among adults infected with COVID-19 in Qatar during 2022 (*N* = 368).

Figure 2 illustrates the prevalence of post-COVID-19 syndrome (PCS) among adults infected with COVID-19 in Qatar during 2022. Notably, approximately 43% of the study participants reported experiencing persistent symptoms for 3 months and more after their initial COVID-19 infection, and these symptoms could not be attributed to any alternative diagnoses.

#### 4.1.3 Frequency distribution of the sociodemographic and health-related characteristics of post-COVID-19 syndrome cases in Qatar during 2022

Table 2 describes the sociodemographic and health-related characteristics of post-COVID-19 Syndrome cases (*n* = 159). The Median age (IQR) of these adults was 37 [IQR: 30.0–45.0] years, with a notable majority of females, accounting for approximately 67.9% (*n* = 108) of the PCS group.

**Table 2:** Frequency distribution of the sociodemographic and health-related characteristics of post-COVID-19 Syndrome cases in Qatar during 2022 (*n* = 159).

Variable	Frequency (*n*)	Percentage (%)
**Age**
18–24	10	6.3
25–39	104	65.4
40 and above	45	28.3
**Median age (IQR)**	37 [IQR: 30.0–45.0]	
**Gender**
Male	51	32.1
Female	108	67.9
**Nationality**
Qatari	16	10.1
Non-Qatari	143	89.9
**Marital status**
Not married	42	26.4
Married	117	73.6
**Education status**
Up to secondary	26	16.4
University and higher education	133	83.6
**Employment status**
Employed	122	76.7
Not-employed	37	23.3
**Perceived Sufficiency of monthly income**
Not enough at all	33	20.8
Just barely enough	31	19.5
Enough	85	53.5
More than enough	10	6.3
**Household crowing index**
Less than 1	24	15.1
1−2	121	76.1
More than 2	14	8.8
**BMI**
Normal weight	38	23.9
Obese/overweight	121	76.1
**Required hospital admission.**
Yes	9	5.7
No	150	94.3
**Required ICU admission (*n* = 9)**
Yes	1	11.1
No	8	88.8

Additionally, most PCS cases were non-Qatari, constituting about 89.9% (*n* = 143) of the group, and most were married (73.6%; *n* = 117), highly educated (83.6%; *n* = 133), and employed (76.7%; *n* = 122).

Moreover, more than half of PCS cases reported having enough perceived monthly income (53.5%; *n* = 85), and a significant proportion (76.1%; *n* = 121) had a household crowding index between 1 and 2.

Regarding health-related features, it’s worth noting that a significant proportion of PCS participants were either obese or overweight, accounting for approximately 76.1% (*n* = 121) of the cases. Additionally, a small percentage of individuals with PCS required hospital admission (5.7%; *n* = 9), and among these cases, only one person (11.1%) required intensive care unit (ICU) admission as elaborated in Table 2.

**Figure 3:**
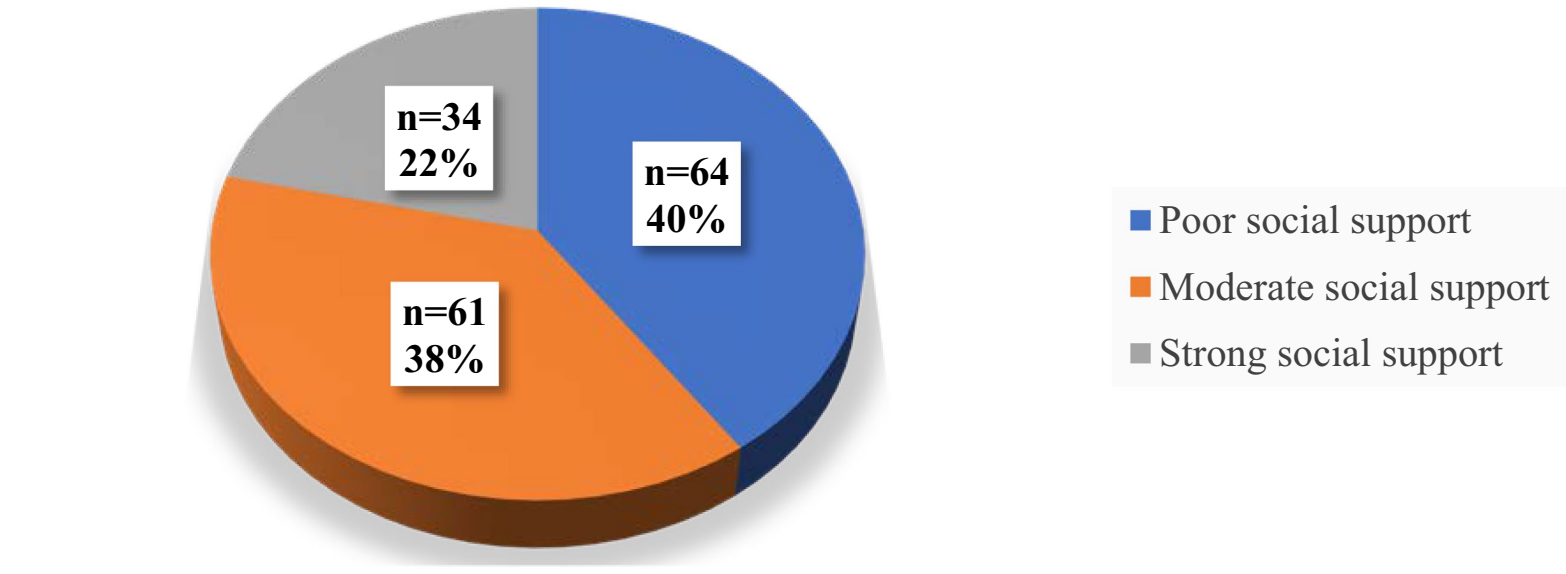
Level of social support among adults with PCS in Qatar during 2022 (*n* = 159).

Regarding the level of social support among PCS patients during 2022. A notable finding was that more than three-quarters of the PCS participants reported having poor to moderate levels of social support, with 40.3 and 38.4%, respectively as shown in Figure 3.

**Figure 4:**
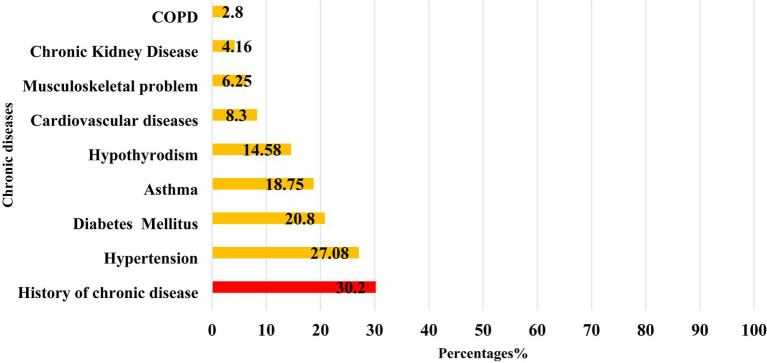
The distribution of common chronic diseases reported among post-COVID-19 syndrome cases in Qatar during 2022 (*n* = 159).

Among the 159 PCS patients, 30.2% (*n* = 48) had a pre-existing chronic health condition. Hypertension was the most frequently reported chronic illness among them, affecting 27.08% of PCS patients, followed by diabetes mellitus (20.8%), asthma (18.75%), and hypothyroidism (14.58%), as shown in Figure 4.

#### 4.1.4 Persistent symptoms among post-COVID-19 syndrome cases in Qatar during 2022.

**Figure 5:**
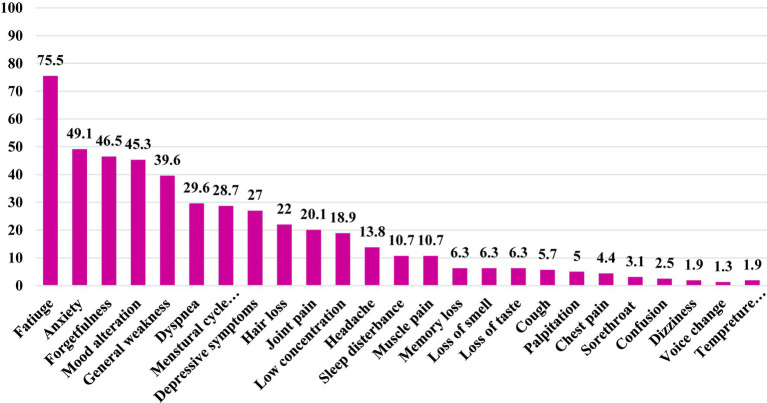
Frequency distribution of the common reported persistent symptoms among adults with post-covid-19 syndorme in Qatar during 2022 (*n* = 159).

Among PCS patients, Fatigue was the most prevalent persistent symptom, accounting for 75.5% of cases, followed by anxiety (49.1%), forgetfulness (46.5%), mood alterations (45.3%), general weakness (39.6%), dyspnea (29.6%), depressive symptoms (27.0%), joint pain (20.1%), reduced concentration (18.9%), and sleep disturbances (10.7%), as shown in Figure 5.

#### 4.1.5 Potential determinants associated with post COVID-19 syndrome

##### 4.1.5.1 Sociodemographic and health-related characteristics associated with post-COVID-19 syndrome

Table 3 shows the association between the sociodemographic characteristics and post-COVID-19 syndrome participants. Several statistically significant risk factors were associated with PCS cases, including gender, educational level, and social support.

**Table 3:** The association between socio-demographic characteristics and post-COVID-19 syndrome cases in Qatar during 2022 (*N* = 368).

	Positive PCS group (*n* = 159)		Negative PCS group (*n* = 209)		
Variable	*n*	(%)	*n*	(%)	*p*-value
**Age**
18–24	10	6.2	18	8.6	0.019*
25–39	104	65.4	106	50.7	
40 and above	45	28.3	85	40.6	
**Gender**
Male	51	32.1	115	55.1	0.001***
Female	108	67.9	94	44.9	
**Nationality**
Qatari	16	10.1	21	10.1	1
Non-Qatari	143	89.9	188	89.9	
**Marital status**
Not married	42	26.4	61	29.2	0.561
Married	117	73.6	148	70.8	
**Education status**
Up to secondary education	26	16.4	60	28.7	0.006**
University/higher education	133	83.6	149	71.3	
**Employment status**
Employed	122	76.7	171	81.8	0.242
Not-employed	37	23.3	38	18.2	
**Perceived monthly income**
Not Enough at all	33	20.8	41	19.6	
Enough	116	73	163	78	0.155
More than enough	10	6.2	5	2.4	
**Household crowding index**
Less than 1	24	15.1	51	24.4	
1−2	121	76.1	139	66.5	0.081
More than 2	14	8.8	19	9.1	
**Level of social support**
Poor social support	64	40.3	52	24.8	
Moderate social support	61	38.3	92	44.1	0.005**
Strong social support	34	21.4	65	31.1	

Level of social support was measured by the Oslo Social Support Scale-3. *p < 0.05; **p < 0.01, ***p < 0.001.

Among the PCS cases, the middle-age group (25–39 years) exhibited a significantly higher prevalence (49.5%; *n* = 104) compared to both younger (35.7%; *n* = 10) and older (34.6%; *n* = 45) age groups, with a *p*-value of 0.019.

The PCS group comprised 30.7% males (*n* = 51) and 53.5% females (*n* = 108), whereas the non-PCS group had 69.3% males (*n* = 115) and 46.5% females (*n* = 94). The female gender exhibited a significantly higher proportion in the PCS group than the non-PCS group, with a *p*-value of 0.00001.

Additionally, university and higher level of education was significantly more prevalent in the PCS group (47.2%; *n* = 133) compared to those with up to secondary education (30.2%; *n* = 26), with a *p*-value of 0.006. Furthermore, the poor level of social support was significantly higher among PCS participants (55.2%; *n* = 64) than non-PCS participants (44.8%; *n* = 52), with a *p*-value of 0.005.

However, no statistically significant differences were observed between the two groups concerning nationality, marital status, employment status, perceived monthly income, and household crowding index as illustrated in Table 3.

##### 4.1.5.2 The association between health-related characteristics and post-COVID-19 syndrome

Table 4 illustrates the association between health-related characteristics and adults with Post COVID-19 Syndrome. Our observations indicated no significant disparities in obesity proportion, chronic disease history, hospital admissions, and ICU admissions, between the PCS groups.

**Table 4:** The association between health-related characteristics/COVID-19 vaccination status and adults with post-COVID-19 syndrome in Qatar during 2022 (*n* = 368).

	Positive PCS group (*n* = 159)		Negative PCS group (*n* = 209)		
Variables	*n*	(%)	*n*	(%)	*p*-value
**BMI**
Obese	121	76.1	151	72.2	0.405
Normal weight	38	23.9	58	27.8	
**History of chronic diseases**
Yes	48	30.2	52	24.8	0.257
No	111	69.8	157	75.2	
**Hospital Admission**
Yes	9	5.6	11	5.3	0.868
No	150	94.4	198	94.7	
**ICU Admission among hospitalized patients (*n* = 20)**
Yes	1	11	1	9	1
No	8	89	10	91	

ICU, intensive care unit; SD, standard deviation; *n*, frequency; %, percentage; CI, confidence. *p < 0.05; **p < 0.01; ***p < 0.001.

However, it was worth noting that obesity appeared slightly more prevalent among individuals with PCS than the non-PCS group (76.1% vs. 72.2%). However, this difference was not statistically significant.

Regarding chronic diseases, the proportion of individuals with a history of chronic illness was higher among the PCS group compared to those with such a history in non-PCS group (30.2% vs. 24.8%) as shown in Table 4.

#### 4.1.6 Sociodemographic and health-related risk factors of post-COVID-19 syndrome

We carried out a multivariable logistic regression model to explore the potential risk factors of post-COVID-19 syndrome characterized by the persistence of symptoms for 3 months and above. The model exhibited statistically significant when compared to the null model and was a good fit. The model’s variables were selected based on clinical and statistical significance. Variables with *p*-values of ≤0.25 in the univariate analysis were included in the regression model.

As delineated in Table 5, our analysis revealed that gender, university, and high educational level, and poor level of social support emerged as significant risk factors of PCS.

**Table 5:** Sociodemographic risk factors of post-COVID-19 Syndrome among adults infected with COVID-19 in Qatar during 2022 using simple (univariate) and multivariable logistic regression analyses (*N* = 368).

Variable	Unadjusted OR (95%CI)	*p*-value	*F* test *P*-value	Adjusted OR (95%CI)	*p*-value
**Age**
18–24	Reference			Reference	
25–39	1.766 [0.779–4.006]	0.173	0.019*	1.566 [0.55–4.481]	0.401
40 and above	0.953 [0.406–2.237]	0.912		0.895 [0.298–2.68]	0.843
**Gender**
Male	Reference			Reference	
Female	2.591 [1.685–3.984]	0.00001***	0.00001***	2.588 [1.58–4.225]	0.0003***
**Nationality**
Qatari	Reference				
Non-Qatari	0.998 [0.503–1.982]	0.996	0.996		
**Marital status**
Not married	Reference				
Married	1.148 [0.7241.822]	0.558	0.558		
**Education status**
Up to secondary education	Reference			Reference	
University/higher education	2.060 [1.22–3.452]	0.006**	0.006**	2.235 [1.256–4.28]	0.011*
**Employment status**
Not-employed	Reference			Reference	
Employed	0.733 [0.441–1.219]	0.231	0.231	0.874 [0.449–1.70]	0.692
**Perceived monthly income**
Not Enough at all	Reference			Reference	
Enough	0.884 [0.527–1.482]	0.640	0.176	0.635 [0.343–1.17]	0.149
More than enough	2.485 [0.773–7.984]	0.126		1.628 [0.408–6.49]	0.490
**Household Crowding index**
Less than 1	Reference			Reference	
1–2	1.850 [1.075–3.184]	0.026	0.084	1.863 [0.984–3.53]	0.056
More than 2	1.566 [0.673–3.641]	0.298		1.362 [0.510–3.64]	0.537
**Level of social support**
Strong social support	Reference			Reference	
Moderate social support	1.268 [0.749–2.145]	0.377	0.005**	1.268 [0.942–2.14]	0.233
Poor social support	2.353 [1.353–4.091]	0.002**		2.451 [1.553–4.13]	0.002**
**BMI**
Normal weight	Reference			Reference	
Obese/Overweight	1.223 [0.762–1.964]	0.405	0.405	1.375 [0.811–2.33]	0.237
**History of chronic disease**
No	Reference			Reference	
Yes	1.306 [0.823–2.07]	0.257	0.257	1.333 [0.74–2.394]	0.335
**Hospital Admission**
No	Reference				
Yes	1.080 [0.43–2.67]	0.868	0.868		
**ICU Admission among hospitalized patients (*n* = 20)**
No	Reference				
Yes	1.250 [0.067–23.25]	0.881	0.881		

*p < 0.05; **p < 0.01; ***p < 0.001.

Females were found to be 2.5 times more likely to develop PCS in comparison to males, as evidenced by some adjusted odds ratio (AOR) of 2.58 (95% confidence interval [CI]: 1.58–4.225), with a *p*-value of 0.0001.

Those individuals with a university or high educational level were approximately two times more likely to experience PCS than those holding up to secondary education, with an AOR of 2.2 (95% CI: 1.256–3.98) and a *p*-value below 0.006.

Furthermore, the likelihood of developing PCS demonstrated an inverse relationship with the level of social support. Individuals with poor social support exhibited significantly higher odds of developing PCS than those with strong or moderate social support, with adjusted odds ratio (AOR) of 2.451 (95% CI: 1.553–4.13, *p* = 0.002) as illustrated in Table 5.

## 5 Discussion

This study aimed to estimate the prevalence of post-COVID-19 syndrome (PCS) and explore the determinants and clinical characteristics of PCS in the adult population of Qatar in 2022. Additionally, the study examined the association between PCS and vaccination history in these individuals.

A comprehensive systematic review conducted in 2021 reported that PCS prevalence ranging from 5 to 80% (10).

In the current study the prevalence of PCS was 43.2% (*n* = 159). This prevalence was notably higher than a prospective observational study conducted among 1,234 adults in Northern India during 2020–2021, where PCS prevalence was 10% (*n* = 122) among patients with persistent symptoms beyond 12 weeks (26). In contrast, other studies in Italy and Germany, Spain, Ecuador, and Belgium reported high of PCS prevalence of (69, 61.9, 56.9, 52.3, and 48%) respectively (11–14, 27). These variations in the prevalence of post-COVID-19 syndrome across different regions of the world might attributed to the socioeconomic characteristics of the population and the specific definition used for PCS duration (10).

The relationship between sex and post-COVID-19 syndrome has been a subject of debate in the available literature, with some studies reporting a strong association between PCS and female sex (11), while others found no sex association (28).

In the present study, PCS was more frequently observed among females (67.9%) than males (32.1%). The Median age (IQR) of adults with PCS was 37 [IQR: 30.0–45.0] years. Notably, most PCS cases were highly educated (83.6%) and employed (76.7%). These findings align with a comparative study in Saudi Arabia, which also found a high prevalence of PCS among middle-aged individuals (mean 36.2, SD± 7.6 years), females (58.7%), those with higher education (68%), and employed participants (64%) (29). Moreover, studies from Switzerland, Italy, Ecuador, United States, and Belgium also observed a predominance of PCS among females, high education level, and employed participants (11, 14, 27, 30, 31). These findings could be related to the risk of exposure to positive COVID-19 cases among employed individuals compared to their non-employed. During the pandemic, individuals in various occupational positions, including frontline workers, police officers, teachers, and others, were required to continue working, heightening their risk of exposure to the virus and subsequently increasing their susceptibility to developing PCS symptoms (32, 33). However, these findings contradict a study in China, where PCS was more common among males and older individuals. This difference could be attributed to variations in the targeted population, with the China study comprising mainly males (53%), and older individuals with median age 59.0 years (IQR 49·0–67·0) (34). Additionally, the discrepancies in these findings regarding gender have been attributed to several factors such as ethnicity, and socioeconomic conditions.

Moreover, the higher prevalence of PCS among women might be attributed to the role of hormones in immune responses, perpetuating the hyper inflammatory status of the acute phase even after recovery. Additionally, differences in serum SARS-CoV-2 IgG antibody concentrations in the early stages of the disease between men and women patients could influence the outcome of COVID-19 (35).

On the other hand, our study could not find an association between socioeconomic level and PCS, as more than half of PCS participants (53.5%) perceived enough monthly income. This finding was consistent with the Menges D et al. study, as most PCS participants had moderate to high monthly income (30). In contrast, a systematic review showed that the risk of PCS increased with the increased level of socioeconomic deprivation (20), and this might be referred to the geographical distribution of participants and living country.

Nevertheless, in our study, most PCS cases had poor to moderate levels of social support (40.3 and 38.4%), respectively, and the logistic regression revealed a significant association between the level of social support and PCS. Consistent with a study conducted in China among 5,982 medical students found that Students with low or medium-level social support had a higher risk of experiencing PCS symptoms than those with high-level social support (36). This result might be attributed to the person’s relationship with other people and surroundings, as social support is one of the coping mechanisms that help people during their illness, loneliness, and during a crisis such as the COVID-19 pandemic. Having a good relationship with others, close to family, friends, or relatives, and finding someone to talk with him are all improve a person’s mental and physical health (36–38).

In our study, 30.2% of PCS patients had comorbidities, including hypertension (27.1%) and diabetes (20.8%). However, none of these conditions were associated with PCS. There was no significant link between hospital admissions and PCS, possibly due to the prevalence of non-communicable diseases and sedentary lifestyles in Qatar’s population, making comparisons inconclusive (39).

Our findings align with previous longitudinal studies such as Tenforde et al. and Petersen et al., which also reported that PCS can affect non-hospitalized individuals with COVID-19, regardless of chronic diseases like hypertension or diabetes. These similarities may be due to the study participants’ age and severity of illness (40, 41).

In our study, the most prevalent persistent symptom among PCS patients was fatigue, affecting 75.5%. This aligns with the findings of other studies, such as Orrù et al. (74%), and Tabacof et al. (82%) (42, 43). However, our study showed a higher percentage of fatigue than Lemhöfer et al. (37.5%), and Izquierdo-Condoy et al. (8.4%) which might be attributed to differences in follow-up duration and case severity (12). Furthermore, selected participants in the Lemhöfer et al. study, which included only mild and moderate COVID-19 cases, could explain the lower percentage of fatigue observed compared to other studies (12, 26, 28, 29, 40, 42, 43).

The evidence showed that fatigue was the most common persistent symptom reported among patients with PCS, which possibly linked to immune mechanisms affecting the brain, neurotransmitter imbalances, and underlying health condition (44, 45). However, our study also reported other prevalent symptoms, including anxiety (49.1%), forgetfulness (46.5%), mood alteration (45.3%), general weakness (39.6%), dyspnea (29.6%), depressive symptoms (27.0%), joint pain (20.1%), low concentration (18.9%), and sleep disturbances (10.7%). These findings are consistent with a comprehensive review by Nalbandian et al. in 2021, highlighting the presence of these symptoms in most PCS patients, though the prevalence may vary (46).

However, it is worth noting that the prevalence of these symptoms varied across different studies. For instance, in our study, a substantial proportion of patients experienced general weakness and joint and muscle pain (70.4%), a finding mirrored by Orrù et al., where muscle aches/myalgia affected 61% of their participants (42).

In contrast, the prevalence of anxiety was lower in the Lemhöfer et al. and Huang L et al. studies (24 and 23%, respectively). Similarly, the occurrence of sleep problems was higher in these studies (30.1 and 26%) compared to our findings (10.7%) (12, 34). However, it is worth noting that the Tabacof et al. study reported an even higher prevalence of sleep disturbances, affecting 59% of PCS cases. These variations could be attributed to differences in the severity of cases in the study samples (43).

### 5.1 Study strength and limitation

#### 5.1.1 Strength

This study adopted the WHO case definition for PCS. In addition, it included both mild and severe COVID-19-infected people and encompassed both hospitalized and non-hospitalized patients. Moreover, we had well-trained data collectors who contributed to the data quality and accuracy, enhancing our findings’ overall reliability.

#### 5.1.2 Limitation

Like any cross-sectional study, there is a potential for recall and selection bias. Conducting a telephone survey may introduce biases, as those who did not answer or participate in the interview may differ from those who did in terms of characteristics and symptoms.

## 6 Conclusion and recommendations

### 6.1 Conclusion

In conclusion, the prevalence of post-COVID-19 syndrome was observed to be higher among the adult population, particularly among middle-aged adults with a history of prior COVID-19 infection. Common persistent symptoms included fatigue, dyspnea, general weakness, anxiety, depression, forgetfulness, and difficulty concentrating. Notably, female gender, university/high educational level, and poor level of social support were significant predictors for PCS.

However, obesity or being overweight did not exhibit a significant association with the development of PCS even though a considerable proportion of PCS patients were obese or overweight.

### 6.2 Recommendation

Promoting public awareness about the “post-COVID-19 Syndrome” and its possible duration and persistent symptoms is crucial so that individuals who experience such symptoms seek appropriate guidance and support. Moreover, policymakers should develop targeted interventions to address PCS and to include PCS as a distinct diagnosis within healthcare guidelines to ensure that individuals affected by this condition receive the specialized care and attention they require.

## Data availability statement

The raw data supporting the conclusions of this article will be made available by the authors, without undue reservation.

## Ethics statement

The studies involving humans were approved by Primary Health Care Corporation Ethical Committee (PHCC-IRB); under protocol number (PHCC/DCR/2022/06/31). The studies were conducted in accordance with the local legislation and institutional requirements. The ethics committee/institutional review board waived the requirement of written informed consent for participation from the participants or the participants’ legal guardians/next of kin because Telephone interview, verbal consent was obtained from the participant.

## Author contributions

NA: Conceptualization, Data curation, Formal analysis, Investigation, Methodology, Project administration, Resources, Software, Supervision, Validation, Visualization, Writing – original draft, Writing – review & editing. MB: Formal analysis, Methodology, Writing – review & editing. MIB: Data curation, Methodology, Supervision, Writing – review & editing. MA-K: Data curation, Methodology, Supervision, Writing – review & editing. AA-K: Data curation, Methodology, Supervision, Writing – review & editing. NS: Conceptualization, Data curation, Formal analysis, Methodology, Supervision, Validation, Writing – original draft, Writing – review & editing.
